# Feedback on dental implants with dynamic navigation versus freehand

**DOI:** 10.6026/97320630019290

**Published:** 2023-03-31

**Authors:** Pragati Nirula, Sahana Selvaganesh, Thiyaneswaran N

**Affiliations:** 1 Department of Implantology, Saveetha Dental college & Hospitals, Saveetha Institute of medical & Technical Sciences (SIMATS), Saveetha University, Chennai 600077, India

**Keywords:** Dynamic navigation, Freehand surgery, Guided implant surgery, Cone beam Computed Tomography, PROM's

## Abstract

The Aim of the study is to understand and identify if Dynamic Navigation (DN) has an upper hand to provide ease and comfort to the patients during and after surgery. 60 patients requiring 120 Implants, were randomly allocated in 2 groups (Group 1- Freehand surgery, Group 2- Dynamic navigation surgery) requiring dental implant therapy. Patients in both the groups were given a Patient satisfaction questionnaire assessing the following domains: comfort, fear, prior experience with robotics, dental anxiety and pain perception. Patient related Experience measures (PREM's) were assessed using the following domains: experience with robotics, pain perception and comfort of using various instrumentations during the surgery. The pre and post CBCT were compared using EVALUNAV application of the Navident software. There was significant difference (p<0.05) between the two groups in the patient outcomes pertaining to the future of robotics in medicine (group 1: 2±1.259, group 2 :4.8±0.484) and Patients reliability to a specific procedure due to the less surgical time taken (group 1:3.3±0.651, group 2: 4.63±0.556). The post-operative pain between the groups was also assessed with VAS scale.

## Background:

In the last decade, the digital revolution has transformed the way dentistry works. With the advent of intra-oral scanners, face scanners, cone beam computed tomography, 3D printers and CAD CAM milling units, an edentulous patient visiting a dental clinic can walk away with a tooth in his mouth within an hour. Many software programmes are used to accurately plan implant positions and fabricate a static guide for implant placement. The static guide's STL (Stereolithography) file can be 3D printed and delivered to the patient's mouth. It is also possible to fabricate the crown before the first stage implant surgery [1].This is how the protocol for static guides works. Dentistry has progressed even further, adopting the most recent technology, Dynamic real-time navigation, which allows the operating doctor to work on the patient with extreme precision. The precise placement of the dental implants can be planned. Teeth loss limits chewing function and, in many cases, causes aesthetics to suffer. These limitations are intended to be addressed by implant prosthetic restorations. To accomplish this, the implants must be placed correctly to ensure a long-lasting and predictable prosthetic restoration. The available bone can be optimally utilised with three-dimensional (3D) prosthetic-based planning. Such planning can be carried out with the help of computer-assisted surgical procedures. Real-time navigation is a dynamic process, whereas template-guided placement is a static procedure. The traditional method still employs freehand implant placement [4]. In addition to achieving an optimal implant position based on prosthetic principles, implantation must avoid damage to adjacent anatomical structures [5]. The three methods for implant placement in patients are free hand, static guides, and dynamic navigation [1]. The implant placement in a static guided system can be done without any additional oral aids and follows the same protocol as free hand implant placement, whereas dynamic navigation systems require the unit to be present inside the operating theatre, which can increase anxiety in the patients receiving treatment [6]. Dynamic Navigation is a system that allows the surgeon to visualise the surgery in real time, developing implant sites on virtual drills while the implant drills are in use. Multiple views are displayed at the same time, displaying the depth, angulation deviations, proximity to adjacent structures, and handpiece orientation. Dynamic navigation procedures have three stages: patient implant diagnostic appointments, virtual treatment planning, and dental implant surgery. The ability to simultaneously see where the drilling is done with the CBCT, throughout the procedure is a significant advantage of dynamic navigation surgery over static guides [7]. While the implant surgeon is focused on the monitor, the dental assistant monitors the surgical site. The virtual treatment plan, unlike a static guide case, can be changed at any time during the procedure. If the plan required modification, a static guide would have to be aborted and either cancelled or proceeded freehand. A dynamic guide system does not obstruct drill irrigation, whereas static guides have been shown to increase heat production in the surrounding bone [2]. The benefits of free-hand implant placement include eliminating the need for a guide and lowering the cost of making the guide [8]. There are some drawbacks to free-hand implantation. First, clinical decisions about implant placement will be based on visualization of the clinical condition provided by cast and radiography information. The second limitation is that this method takes longer than the surgical guide method because free-hand implant placement necessitates thought and planning. Patient related outcome measures (PROMs) are used to evaluate a procedure's patient-related outcomes [9]. PROMs are validated questionnaires that are standardised to be completed by patients before and after surgery to provide information about the patient's health status, level of impairment, and health-related quality of life [10]. The efficacy of the patient's perception is measured through the patient's perspective. Questionnaires are given to patients both before and after surgery in order to compare the outcomes of the procedure. PREM's are classified as two types-Relational PREM's: This type of PREM identifies the patients experience during the treatment, e.g - did they feel comfortable and Function PREM's? This type of PREM examines more practical issues, such as the facilities available for the treatment. Therefore, it is of interest to understand and identify that dynamic navigation has an upper hand to give ease and comfort to the patients during and after surgery. Null Hypothesis-Dynamic navigation does not help in providing ease and comfort to the patients during and after surgery. Alternate hypothesis-Dynamic navigation helps in providing ease and comfort to the patients during and after surgery.

## Materials & Methodology:

This study was a single-centre, Parallel group, randomised controlled clinical trial. It was structured and reported following the CONSORT guidelines. Our team has extensive knowledge and research experience that has ability to translate into high quality publications [11,12,13, 14,15,16, 17,18,19, 20]. The department study was approved by the institutional review board. The study was conducted from October 2022 to February 2023 in the Department of Oral Implantology at Saveetha dental college, Chennai, India.

## Patients were randomly allocated into two groups:

Group 1: Brain Guided/ Free hand surgery

Group 2: Navident associated surgery

Sixty patients who met the inclusion criteria (that is, at least 20 years of age who require adjacent implant placement are considered) accepted to participate in the trial and were randomly allocated to two groups, according to the dental implant placement protocol, by means of block randomization.

Inclusion criteria: Patients who required two adjacent parallel implants

Exclusion criteria: Patients who required full mouth rehabilitation (FMR's)

The included subjects were required to undertake the self-administered questionnaire concerning: 1) Pre-operative expectations ( that is, five items evaluating patient's perception and expectations concerning the planned surgery using a 5-point Likert scale),2) Post-Operative pain intensity evaluation (that is, pain intensity assessment on a continuous visual analogue scale VAS-SCALE till 48 hours), 3) Post-operative PROMs ( that is, post-operative symptoms experience and overall patient satisfaction using 5-point Likert scale)

## Surgical protocol followed for freehand surgery (Group 1):

Pre-operatively during the treatment planning phase, the CBCT DICOM data and intra-oral scan of the patient (STL file) was merged and the planning of the implant was done under the dynamic navigation software. On the day of surgery, a preoperative questionnaire was filled by the patient. Local anaesthesia (lignocaine with 2% Adrenaline) was administered and an incision was made. Full thickness mucoperiosteal flap elevation was done. Initial drilling was done and then the precision was checked by the position indicating device with x-ray. Subsequent sequential drilling was done and then Implant of the planned dimensions was placed. Post implant placement instructions were given and post CBCT was done. Post-operative questionnaire asked to the patient and VAS scale for pain monitoring was assessed immediately post-surgery and after 48 hours of the surgery. The pre and post CBCT's were compared with EVALUNAV in the Navident software.

## Surgical protocol followed for Dynamic Navigation surgery (Group 2):

## Patient implants diagnostic appointment:

The diagnostic appointment is scheduled for 30 minutes and is usually completed 2 weeks prior to the day of surgery. During this appointment, the patient is subjected to CBCT. Once the CBCT was verified to be free of motion distortion; the intra-oral scanning of the patient which gives an out as STL data.

## Virtual treatment planning:

The Digital Imaging for Communication in Medicine (DICOM) data set was uploaded into the Dynamic navigation (Navident) treatment planning software. If a lower posterior implant was to be planned, the inferior alveolar nerve was mapped. The software allows for a virtual wax-up of the proposed restoration and the ability to freely move it into position and change its dimensions. Once the virtual wax-up was complete, implant planning was commenced. The dental implants were planned in a generic fashion, allowing their height and width to be adjusted.

## Dental implant surgery:

On the day of surgery, a preoperative questionnaire was filled by the patient. Local anaesthesia was administered and then the jaw trackers were fitted in the jaw that was to be operated on. If it is the maxillary arch, a head tracker was placed. Once the patient is comfortable, trace registration is carried out with the selected points on the teeth and the accuracy is assessed by placing the tracer tip in the occlusal, buccal and lingual sites. The accuracy should not exceed or recede 1mm, which will in turn cause an error in the implant positioning in three Dimensional axis. After the calibration of the jaw to the navident system is complete, the drill tag which is fitted on to the handpiece is calibrated to the computer and the axis of the handpiece and the drills are calibrated. While drilling the surgeon is guided to the site and a dart appears on the screen that indicates the angular deviation and Mesio-distal and Bucco-lingual deviations. Once the sequential drilling and osteotomy sites are prepared for all the implant sites, the particular implants are calibrated and then placed into the site. Pre and post CBCT were compared with EVALUNAV in the Navident software.

## Results:

60 participants (mean age= 48 years) corresponding to 120 Implants placed were evaluated. Most of the subjects had missing teeth in anterior sites (20%) and posterior sites (80%) with respect to adjacent parallel Implants. Nevertheless, there was a significant difference between inter-group surgical time (Mean time Group 1=60 mins, Group 2= 28.3 mins) with p<0.05. Concerning the Preoperative patients' perspectives on dental implant intervention, most participants believed that implants would permit chew (94%), appearance (91%) to be close as with natural dentition. There was a significant difference between the groups regarding the both the amount of time expected to be in pain post-operatively (p=0.03). In the Patient satisfaction questionnaire, Q4, Q5 and Q9 (Pre-surgery Questionnaire) had significant difference (p<0.05) between both the groups i.e, Freehand group and the Dynamic navigation group. There was a significant difference between the groups regarding both the amount of time expected to be in pain post-operatively (p=0.03) when the VAS scale values were assessed. Q3 and Q5 ( Post-surgical questionnaire) also had a significant difference as most of the people from the freehand group were not comfortable with the machines that were kept in the OT while doing the surgery & 96% of the patients of the dynamic navigation group believe that Robotics is the future forward in dentistry.

In the Post-Surgical questionnaire; 96.7% patients from group 1( Freehand group) and group 2(Dynamic navigation) were comfortable during the procedure,90% patients from group 1 and 83.3% from group 2 were not anxious during the procedure, 60% of patients from group 1 were not comfortable with the machine kept in the OT whereas 93.4% from group 2 were comfortable with the machines kept, 83.3% patients from group 1 and 90% from group 2 did not feel any pain during the procedure,76.6% patients from group 1 and 100% from group 2 believe that Robotics is the future forward.,96.6% patients from group 1 and group 2 felt comfortable if the doctor was not looking at the site.

## Discussion:

DN technology is more environmentally friendly because it is entirely virtual and does not require templates or analogous impressions. With the application of new technology, there is a learning curve for all levels of technological comfort. DN has been reported to be at least as accurate as tooth-supported surgical guide systems for implant drilling and placement, and significantly more accurate than the free-hand approach, despite the fact that now the literature is still limited. Because the system is open-source, it's possible to integrate it with any implant system. A considerable advantage of the DN is that the computer aided treatment planning helps in the proper visualisation of the bone and the adjacent structures. As this helps in the merging of the clinical data with that of the radiographic data, it helps the surgeon to be ultimately precise. This technology helps in planning a flapless approach for implant placement and also reduces the time taken for placing 2 adjacent parallel implants. By these means DN can reduce the chair side time for the patient including the impression making, mock-up of the missing teeth and planning the case prosthetically. From the operator point of view more research is needed to determine whether dynamic navigation or static guidance is superior for implant placement. Because surgery is not performed with a thermoplastic stent in place, Trace Registration technology provides a completely digital workflow. It avoids the time-consuming and technique-critical step of fabricating a custom stent prior to surgery, reduces the need for a second CBCT scan with fiducial markers, and improves surgical site access. This technology has increased DN's efficiency and applicability. The most significant advantage of DN is that it allows for continuous accuracy checks during surgery, which is not possible with a static approach.

## Conclusion:

Placing dental implants by Dynamic navigation and conventional brain-guided surgery had a significant difference in pre-operative perspective of patients towards robotics & procedure being minimally invasive. Immediate post-operative pain perception, levels of comfort were better with dynamic navigation and the future of robotics in dentistry also had a huge welcome among patients as they were intrigued as they can visualise and look at the screen and understand what the procedure was and how and where the implants were placed in their jaw.

## Ethical approval:

All procedures performed in the studies involving human participants were under Ethical standards of the institution and with the Helsinki declaration and its later amendments Ethical standards.

## Funding:

This study is not a funded project.

## Figures and Tables

**Table 1:** Representing the significance for various questions between Dynamic navigation group and free hand brain guided surgery.

**PREOPERATIVE QUESTIONS**	**Groups**	**Mean ± Std**	**Sig(2-tailed)**
Q1- Are you scared of the procedure explained?	1	3.97± 1.326	0.753
	2	4.07±1.112	0.753
Q2-Do you think implant placement will help your mastication/ Aesthetics / Phonetics?	1	4.03±0.49	0.595
	2	3.9±1.269	0.593
Q3-Do you feel anxious about the outcomes of the procedure?	1	3.7±1.317	0.754
	2	3.6±1.133	0.754
Q4-Do you think Robotics can do a better job than human doctors?	1	2±1.259	0.00*
	2	4.8±0.484	0.00*
Q5-Do you want the procedure to be minimally invasive?	1	3.3±0.651	0.00*
	2	4.63±0.556	0.00*
Q6-Even if Invasive, do you think the outcome is what matters?	1	4.13±0.507	0.319
	2	4.27±0.521	0.319
Q7- How soon do you wish to go back to normalcy?	1	3.83±0.592	0.479
	2	3.97±0.89	0.498
Q8- Was your prior dental experience with robotics Bad?	1	3.63±0.615	0.108
	2	3.23±1.19	0.11
Q9- Do you wish the surgical time to be as minimal as possible?	1	3.17±0.379	0.00*
	2	5±0	0.00*
Q10- VAS-SCALE(PAIN MONITORING TILL 48 hrs):	1	1.73±1.437	0.76
	2	0.23±0.568	0.03*
POST OPERATIVE QUESTIONS	1	1.03±0.183	0.561
Q11-Did you feel comfortable during the procedure?	2	1.07±0.254	0.562
Q12- Were you anxious during the procedure?	1	1.9±0.305	0.456
	2	1.83±0.379	0.456
Q13- Were you comfortable with the various machines in the surgical OT?	1	1.8±0.407	0.00*
	2	1.03±0.183	0.00*
Q14- Did you feel pain during the procedure?	1	1.83±0.379	0.456
	2	1.9±0.305	0.456
Q15- Do you think robotics is the future?	1	1.8±0.407	0.00*
	2	1±0	0.00*
Q16-Did you feel comfortable with the doctor not looking at the site on the computer screen during the operating procedure?	1	1.03±0.183	1
	2	1.03±0.183	1
*Significant difference p<0.05
